# Preoperative moderate thrombocytopenia is not associated with increased blood loss for low-risk cesarean section: a retrospective cohort study

**DOI:** 10.1186/s12884-019-2417-1

**Published:** 2019-07-29

**Authors:** Xiaohan Xu, Yuelun Zhang, Xuerong Yu, Yuguang Huang

**Affiliations:** 1 Department of Anesthesiology, Chinese Academy of Medical Sciences & Peking Union Medical College Hospital, Beijing, 100730 China; 2 Central Research Laboratory, Chinese Academy of Medical Sciences & Peking Union Medical College Hospital, Beijing, 100730 China

**Keywords:** Thrombocytopenia, Blood loss, Cesarean section, Blood transfusion

## Abstract

**Background:**

The occurrence of thrombocytopenia is as high as 7–12% in pregnancy, yet minimum platelet count safe for cesarean section remains unknown.

**Methods:**

In this retrospective noninferior cohort study, we consecutively included patients undergoing cesarean section for a period of 6 years in a tertiary hospital and excluded patients at very high risk for excessive hemorrhage. The included patients with preoperative platelet count of 50–100 × 10^9^/L were defined as the thrombocytopenic group. The control group were eligible patients with preoperative platelet count>150 × 10^9^/L, matched to the thrombocytopenic group by age and operation timing in a 1:2 ratio. Mixed effect model was used to analyze the effect of thrombocytopenia based on a noninferiority assumption. The predefined noninferiority delta of bleeding was 50 mL.

**Results:**

There was no significant difference of the calculated blood loss between the thrombocytopenic and the control group (mean difference = 8.94, 95% CI − 28.34 mL to 46.09 mL). No statistical difference was observed in the requirement for blood transfusion, visually estimated blood loss, or the incidence of adverse events between groups. Although there were more patients admitted to intensive care unit (odds ratio = 12, 95% CI 2.69–53.62, *p* = 0.001) in the thrombocytopenic group, most of them required critical care for reasons other than hemorrhage. The thrombocytopenic group had longer length of hospital stay (mean difference = 0.40 days, 95% CI 0.09–0.71, *p* = 0.011), but the difference was considered as clinically insignificant.

**Conclusions:**

Preoperative moderate thrombocytopenia is not associated with increased blood loss, blood transfusion, or occurrence of adverse events in patients undergoing cesarean section in absence of additional bleeding risk.

## Introduction

It has been recommended by guidelines that a preoperative platelet (PLT) count of 50× 10^9^/L or greater is safe for elective major surgery except neurosurgery or ophthalmic surgery involving the posterior segment of the eye [1, 2]. However, the strength of such recommendation is weak due to the very low quality of evidence. Since the bleeding risk may vary enormously among different types of surgery, it is more reliable to evaluate the tolerance of thrombocytopenia for each specific surgery type. To the best of our knowledge, there is scarce convincing evidence indicating the impact of preoperative moderate thrombocytopenia (PLT 50–100 × 10^9^/L) on blood loss for cesarean section (CS).

The occurrence of thrombocytopenia (PLT less than 150 × 10^9^/L) is as high as 7–12% in pregnancy [3]. Excessive blood loss at delivery is one of the leading causes for maternal mortality [4]. As a consequence, there has been considerable concern over the safety of thrombocytopenic patients undergoing CS in the past years. Recent publications indicate that delivery could be uneventful for most patients with idiopathic thrombocytopenic purpura (ITP) and gestational thrombocytopenia (GT) [5, 6]. On the contrary, patients with HELLP (hemolysis, elevated liver enzymes, low platelets) syndrome are at high risk for the sudden development of disseminated intravascular coagulation (DIC) and associated massive hemorrhage [7]. Given the heterogeneous etiology of thrombocytopenia, it is difficult to determine the indication for specific therapy to raise PLT count before CS. Actually, the minimum PLT count acceptable for CS differs greatly among medical centers, ranging from to 50–80 × 10^9^/L [6, 8, 9]. That is where the controversies arose and continued.

In this work, we designed this retrospective noninferior cohort study to evaluate the association of moderate thrombocytopenia with the calculated blood loss during and after CS. We hypothesized that moderate thrombocytopenia does not have clinically significant effect on blood loss.

## Methods

### Study design and population

We included consecutive patients undergoing CS from June 1, 2012 to 31 May 2018 in Peking Union Medical College Hospital, Beijing, China, which is a 2300-bed, university-affiliated, tertiary hospital. To reduce the impact of other factors that may affect blood loss or transfusion requirement, we excluded patients with: (1) abnormal coagulation test, anti-coagulation, or anti-platelet therapy within 1 week prior to surgery; (2) previous history of postpartum hemorrhage (PPH) or platelet dysfunction; (3) abnormal placentation, including placenta previa, vasa previa, placenta accreta, and placental abruption; (4) preoperative spontaneous hemorrhage, active bleeding, or anemia (hemoglobin, HGB<100 g/L); (5) prophylactic platelet transfusion which was defined as platelet transfusion within 7 days prior to CS. The eligible patients whose PLT was 50–100 × 10^9^/L in the last count of blood cell (CBC) test before surgery were defined as thrombocytopenic group. The control group was selected from patients who also met the inclusion and exclusion criteria with preoperative PLT>150 × 10^9^/L. Considering the total number of the control patients and the availability of the matching method, they were matched to cases in the thrombocytopenic group in a 1:2 ratio by age (±5 years) and operation timing (emergency vs. elective).

### Perioperative management

The obstetrician team was consisted of a fellow, a chief resident, and a resident. Surgical techniques were standardized and for all the operations. Most patients underwent neuraxial anesthesia (NA), which was either spinal anesthesia, epidural anesthesia, or their combination. For the concerned about epidural hematoma, NA was contradicted if PLT count was less than 80 × 10^9^/L [10]. General anesthesia (GA) would be initiated when NA was contradicted or not effective, or when there was no sufficient time for performing NA in urgent cases. Patients usually received lactated Ringer’s solution 500–1000 ml and colloid solution 500 ml in the operating room. Crystalloid solution 1500 ml was continued to be intravenously infused after the patients returned to the ward. Oral drinking was allowed 6 h after surgery.

CBC test was routinely performed within 1 week prior to CS, and re-performed in 24–36 h after surgery. To prevent obstetric hemorrhage, a bolus dose of 10 IU oxytocin was given intravenously over 1 min, and another 10 IU dissolved in 500 mL of 0.9% saline solution was infused after the uterus was sutured. RBC was transfused intraoperatively and/or postoperatively if the HGB level was lower than 80 g/L or there were signs for uncontrollable ongoing hemorrhage. Fresh frozen plasma (FFP) and platelet would be transfused together if necessary.

### Data collection and outcomes

Demographic and intraoperative information was collected from electronic anesthesia record, including age, body weight, height, gestational weeks, operation duration, birth weight, and anesthesia method.

The calculated blood loss was the primary outcome. Hematocrit (HCT) in the last preoperative CBC test and in the first postoperative CBC test was recorded. We used the following equation [11] for blood loss calculation.

$$ Calculated\ blood\ loss/ ml= estimated\ blood\ volume/ ml\times precentage\ of\  HCT\  derease $$

$$ Estimated\ blood\ volume/ ml=0.75\times \left[\left( height/m\times 1968.50\right)+\left( weight/ kg\times 55.12\right)\right] $$

$$ Precentage\ of\  HCT\  decrease=\left( preoperative\  HCT- postoperative\  HCT\right)/ preoperative\  HCT $$

For patients who received RBC transfusion during surgery, the blood loss before transfusion can be calculated based on the change between preoperative HCT and pre-transfusion HCT, while similarly the blood loss after transfusion can be calculated by post-transfusion HCT and postoperative HCT. Their total blood loss was the sum of these two parts.

The secondary outcomes were observed over a period of postoperative hospitalization. We extracted the data from medical records about: (1) the requirement for blood transfusion, (2) visually estimated intraoperative blood loss, (3) surgical site bleeding requiring a second invasive therapy (open surgery or interventional embolization), (4) the occurrence of postoperative adverse events (infection, delayed wound healing, cardiac event, cerebrovascular event, thromboembolism event), (5) the requirement for postoperative intensive care unit (ICU) stay, (6) the length of hospital stay, (7) all-cause mortality.

### Statistical analysis

We described the baseline characteristics of the eligible patients using descriptive analysis. Continuous data were expressed as mean (standard difference) (normally distributed data) or median (quartiles) (non-normally distributed data). Patients’ baseline characteristics were compared between groups using the standardized difference and *p* value, where standardized difference smaller than 0.2 or *p* value greater than 0.05 was considered as acceptable deviation between groups. Unbalanced variables at baseline were included in the mixed-effect model to adjust for the potential confounding effect.

The primary outcome of blood loss was analyzed in a noninferior fashion. Based on clinical experience, we used 50 mL as the clinically significant noninferior delta. The hypothesis was that the thrombocytopenic group was non-inferior to the control group for 50 ml blood loss. Considering the matched design between the exposed and the control groups, we used the mixed-effect model to analyze the effect of preoperative thrombocytopenia, in which the dependent variable was the calculated blood loss. Blood measures at individual level were analyzed as lower level of data while the effect of matched pairs was analyzed as higher level of data in the mixed effected model. The mean difference of blood loss between the thrombocytopenic and the control groups and the standard error of the mean difference from the mixed-effect model was used to calculate the two-side 95% confidence interval (CI), which would be compared with the predefined noninferiority delta.

If the upper bound of the noninferior 95% CI was smaller than 50 mL, we rejected the null hypothesis and concluded that the blood loss in the thrombocytopenic group was not larger than in the control group.

The effect of thrombocytopenia on blood loss may be influenced by other confounding factors. American Society of Anesthesiologists (ASA) classification of physical status, body mass index (BMI), gestational weeks, previous uterine scar, neonatal macrosomia, multiple pregnancies, anesthesia method and operation duration were regarded as potential confounders based on previous publications. We checked if their distributions were balanced between the thrombocytopenia and the control groups, and unbalanced factors were included in the adjusted models.

We used the mixed-effect model to compare visually estimated blood loss and the length of postoperative hospital stay between the thrombocytopenic group and the control group due to its non-normal distribution. Conditional logistic regression was used to compare the other binary secondary outcomes between the two groups.

Mixed-effect and conditional logistic regression models were built in R (version 3.4.4) using the “lme4” and “survival” packages. Other analyses were conducted in SPSS (version 13, SPSS, Inc., Chicago, IL, USA).

Since the eligible participants in our center during the study period were limited, we used the sample size in the analysis to calculate the statistical power based on the noninferiority assumption. Considering the clinical relevance, we employed the primary outcome and calculated blood loss as the key outcome in the power calculation. Probability of type I error was set to two-side 0.05. Sample size was 155 in the thrombocytopenic group and 310 in the control group. The noninferiority delta was 50 mL considering the clinical relevance. Based on the above parameters, the power of our analysis was 85.86%.

## Results

### Baseline characteristics

**Fig. 1:**
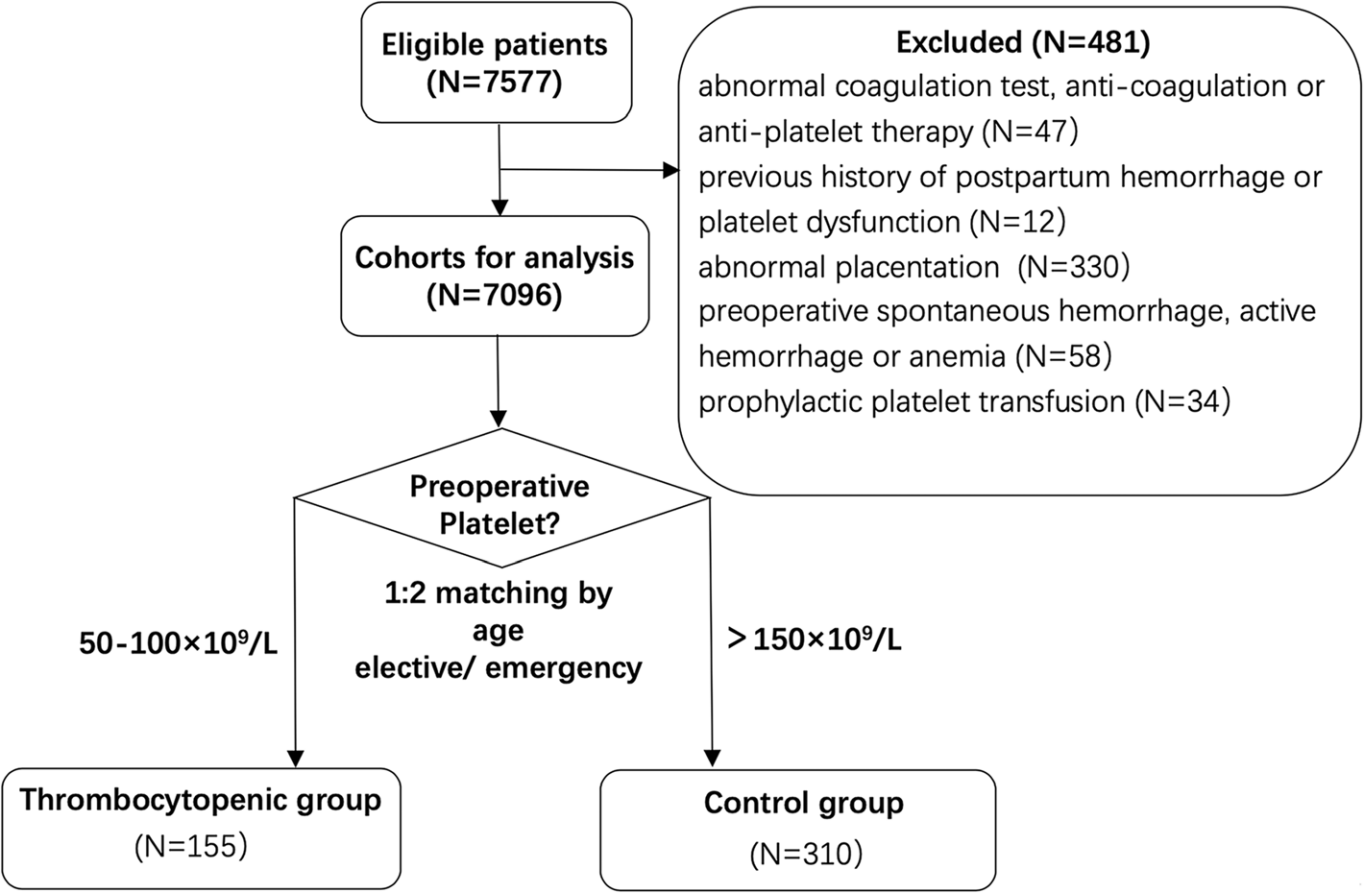
Study design and group allocation. Abbreviation: PLT, platelet

A total of 7577 patients received CS over the six-year study period. 481 of them were excluded for the reasons shown in Fig. 1. Among the remaining 7096 cases, 155 patients had preoperative PLT 50–100 × 10^9^/L and thus were included in the thrombocytopenic group. Matched by a ratio of 1:2, 310 patients with preoperative PLT>150 × 10^9^/L were included in the control group.

The preoperative platelet count was (71.9 ± 13.0) × 10^9^/L in the thrombocytopenic group and (203.6 ± 41.5) × 10^9^/L in the control group. The etiologies for thrombocytopenia were gestational thrombocytopenia (GT) (70, 45.2%), pre-eclampsia (22, 14.2%), idiopathic thrombocytopenic purpura (ITP) (19, 12.3%), systemic lupus erythematosus (SLE) (17, 11.0%), mixed connective tissue disease (3, 1.9%), myelodysplastic syndrome (3, 1.9%), hypersplenism (1, 0.65%) and thrombotic thrombocytopenic purpura (1, 0.65%). The etiology was unknown for the rest of patients. Baseline characteristics between the two groups are displayed in Table 1. We did not find significant difference in age, ASA, previous uterine scar, neonatal macrosomia, multiple pregnancies, operation timing and operation duration between groups, whereas BMI (standardized difference = 0.34, *p* = 0.001), gestational weeks (standardized difference = 0.24, *p* = 0.018) and anesthesia method (standardized difference = 0.63, *p<*0.001) were not balanced in the two groups.

**Table 1:** Baseline Characteristics

	Thrombocytopenic Group (*n* = 155)	Control Group (*n* = 310)	Standardized difference	*p* value
Age (yrs)	31.5 ± 4.5	31.5 ± 4.3	0.01	0.905
BMI (kg/m^2^)	26.2 ± 3.0	27.1 ± 2.8	0.34	0.001^*^
ASA			0.12	0.271
II	134 (86.5%)	280 (90.3%)		
III	21 (13.5%)	30 (9.7%)		
Gestational weeks	37.8 ± 1.4	38.1 ± 1.3	0.24	0.018^*^
Birth weight (g)	3024.3 ± 468.3	3059.6 ± 452.1	0.08	0.438
Previous uterine scar			0.01	1.000
Yes	28(18.1%)	55 (17.7%)		
No	127(81.9%)	255 (82.3%)		
Neonatal macrosomia			0.06	0.716
Yes	7 (4.5%)	18 (5.8%)		
No	148 (95.5%)	292 (94.2%)		
Multiple pregnancies			0.03	0.938
Yes	8 (5.2%)	14 (4.5%)		
No	147 (94.8%)	296 (95.5%)		
Anesthesia method			0.63	<0.001*
NA	120 (77.4%)	302 (97.4%)		
GA	35 (22.6%)	8 (2.6%)		
Operation timing			0.00	1.000
Elective	86 (55.5%)	172 (55.5%)		
Emergency	69 (44.5%)	138 (44.5%)		
Operation duration (min)	60.7 ± 16.1	57.9 ± 14.3	0.07	0.474
Preoperative PLT (×10^9^/L)	71.9 ± 13.0	203.6 ± 41.5	4.29	<0.001*

*Abbreviations*: *BMI* body mass index, *ASA* American Society of Anesthesiologists classification of physical status, *NA* neuraxial anesthesia, *GA* general anesthesia

Values are expressed as mean ± SD or number (percentage) of patients. Patients’ baseline characteristics were compared between groups using the standardized difference and *p* value, where a standardized difference smaller than 0.2 and *p* greater than 0.05 was considered as acceptable deviation between groups. The two groups were balanced in age, operation timing, ASA, the presence of underlying risk factors and operation duration. On the contrary, they were not balanced in BMI, gestational weeks and anesthesia method

### Primary outcome

The calculated blood loss was 417.8 ± 194.6 ml in the thrombocytopenic group and 414.5 ± 182.9 ml in the control group. The coefficient of thrombocytopenia effect in the mixed-effect model was 3.3, indicating that the calculated blood loss in the thrombocytopenic group was 3.3 mL higher than that in the control group (Table 2). According to the formula in the method section, the upper bound of the two-side 95% CI of the calculated blood loss was 37.67 mL, lower than the predefined noninferiority delta (50 mL). We built the following 5 models to examine the independent effect of thrombocytopenia on blood loss. Besides the unbalanced factors— BMI, gestational weeks and anesthesia method, we also include operation duration into models, based on our clinical experiences.

- Model 1: blood loss~ thrombocytopenia
- Model 2: blood loss~ thrombocytopenia + operation duration
- Model 3: blood loss~ thrombocytopenia + operation duration + BMI
- Model 4: blood loss~ thrombocytopenia + operation duration + BMI + anesthesia method
- Model 5: blood loss~ thrombocytopenia + operation duration + BMI + anesthesia method + gestational weeks

**Table 2:** Effect of Thrombocytopenia for the Calculated Blood Loss in the Mixed-effect Model in 465 Patients

Model ^a^	95% confidence interval (ml)
1^b^	−31.06 to 37.67
2 ^b^	−31.09 to 37.69
3 ^b^	−29.32 to 40.28
4 ^b^	−29.05 to 45.17
5 ^b^	−28.34 to 46.09

*Abbreviations*: *BMI* body mass index;

^a^ The reference group in the mixed-effect models was the control group

^b^ Model 1: blood loss~ thrombocytopenia, Model 2: blood loss~ thrombocytopenia + operation duration, Model 3: blood loss~ thrombocytopenia + operation duration + BMI, Model 4: blood loss~ thrombocytopenia + operation duration + BMI + anesthesia method, Model 5: blood loss~ thrombocytopenia + operation duration + BMI + anesthesia method + gestational weeks

The fifth mixed-effect model that includes thrombocytopenia, operation duration, BMI, anesthesia method and gestational weeks yielded a coefficient of 8.94, indicating that the calculated blood loss in the thrombocytopenic group was 8.94 mL (95% CI − 28.34 to 46.09 mL) greater than that in the control group. In summary, the noninferiority assumption was claimed in all the 5 models.

### Secondary outcomes

The descriptive analysis and comparison of secondary outcomes was demonstrated in Table 3. Five patients (3.2%) and seven (2.3%) patients received transfusion in the thrombocytopenic and the control group respectively. Among them, one in the thrombocytopenic group and one in the control group with a calculated blood loss of about 1600 ml received 4 U RBC and 800 ml FFP. Additionally, the former one also received 2 U platelet. Two patients (one for each group) had a calculated blood loss of 1000–1500 ml and received 2 U RBC along with 400 ml FFP. The remaining eight patients only received 2 U RBC. There were two (1.3%) cases of postoperative adverse events in the thrombocytopenic group, including one right heart failure secondary to pulmonary hypertension and one suspected infection (febrile lasting for more than 24 h with no pathogenic evidence). The two (0.65%) cases of adverse events in the control group were suspected infection and venous thromboembolism. Twelve (7.7%) in the thrombocytopenic group and two (0.65%) in the control group went to ICU after surgery, including the four who required RBC and FFP transfusion and the one with heart failure mentioned above. The remaining nine of them were all patients with autoimmune disease from the thrombocytopenic group. They spent 1–3 days in ICU for the concern about the progression of lupus nephropathy or the relapse of lupus encephalopathy. No invasive hemostatic intervention or death was observed in both groups.

**Table 3:** Descriptive analysis and Comparison of Secondary Outcomes

Variable	Thrombocytopenic Group (*n* = 155)	Control Group (*n* = 310)	Estimate (OR or mean difference)	95% CI	*p* value
Blood transfusion	5 (3.2%)	7 (2.3%)	1.55	0.43–5.58	0.505
Visually estimated blood loss	315.8 ± 150.0	297.6 ± 126.6	18.23	−7.07-43.52	0.158
Hemostatic therapy	0 (0%)	0 (0%)	/	/	/
Adverse event	2 (1.3%)	2 (0.65%)	2.00	0.28–14.20	0.488
ICU stay	12 (7.7%)	2 (0.65%)	12.00	2.69–53.62	0.001^a^
Length of hospital stay	3 [3,4]	3 [3,4]	0.40	0.09–0.71	0.011^a^
All-cause mortality	0 (0%)	0 (0%)	/	/	/

*Abbreviations*: *ICU* intensive care unit, *OR* odds ratio, *CI* confidence interval;

Continuous data was expressed as mean (SD) (normally distributed data) or median (quartiles) (non-normally distributed data. Categorical data was expressed as number (percentage). Visually estimated blood loss and the length of postoperative hospital stay was compared by mixed-effect model. Other parameters were compared by conditional logistic regression. ^a^ Significant difference

No statistical difference was observed regarding the requirement for blood transfusion, visually estimated intraoperative blood loss, or the incidence of adverse events between groups (Table 3). However, there were more patients admitted to ICU (odds ratio 12.00, 95% CI 2.69 to 53.62, *p* = 0.001) and longer length of hospital stay (mean difference = 0.40 day, 95% CI 0.09 to 0.71, *p* = 0.011) in the thrombocytopenic group.

## Discussion

In the present study, we respectively reviewed the calculated blood loss, visually estimated blood loss, blood transfusion, invasive hemostatic intervention, postoperative adverse events, ICU stay, hospital stay and all-cause mortality in low-risk CS patients with preoperative PLT 50–100 × 10^9^/L over a period of 6 years in a tertiary medical center. We found that preoperative moderate thrombocytopenia is not associated with increased blood loss, requirement for blood transfusion, or incidence of adverse events.

It has been reported that the leading causes for thrombocytopenia are GT, pre-eclampsia and ITP [12], which are consistent with our data. GT is resulted from hemodilution and increased platelet turnover during pregnancy [13], and requires no treatment as it is not associated with increased bleeding risk or altered delivery mode [10]. Studies in ITP complicating pregnancy demonstrated that severe morbidity or mortality was uncommon, despite the moderate drop of PLT [14, 15]. Hence, there has been a trend towards a more conservative management [16, 17], even without much information about the blood loss at delivery in thrombocytopenic pregnant patients.

Guidelines recommend a PLT threshold of 50 × 10^9^/L prior to major surgery and 100 × 10^9^/L for neurosurgery and ophthalmic surgery [1, 2, 18, 19]. However, it has been found that a preoperative platelet count of 50–100 × 10^9^/L is associated with increased requirement for blood transfusion and 30-day mortality after non-cardiac surgery [20]. Since the minimum PLT count safe for CS remains unclear, decisions on whether to raise PLT count before CS are often based on physicians’ clinical experience rather than high-quality evidence. Thromboelastography has been indicated to be a good predictor of bleeding risk in patients with severe thrombocytopenia (PLT<10 × 10^9^/L) caused by hematologic malignancies [21]. Nevertheless, its predictive value in patients with PLT 50–100 × 10^9^/L is still open to doubt, and the high cost may limit its use.

Our findings elucidate that patients with preoperative moderate thrombocytopenia had no more blood loss, blood product transfusion, or postoperative adverse events than the ones with normal PLT count. Based on these findings, we could cautiously speculate that raising PLT count to above 100 × 10^9^/L before low-risk CS might bring little obvious benefits to patients without massive hemorrhage. Preoperative PLT transfusion is commonly used to rapidly raise PLT count in thrombocytopenic patients. However, such prophylactic transfusion might be unnecessary for CS patients with PLT 50–100 × 10^9^/L in absence of additional bleeding risk. In this way, a series of transfusion-associated risks could be reduced, including febrile, allergic reactions, arrhythmia, transmissible infections, hemolysis, auto-antibody production, transfusion related acute lung injury and immunomodulation [2, 22].

The blood loss was calculated based on pre- and post-operative HCT. Its inaccuracy mainly results from the change of circulating blood volume [23], which could be minimized in our study by applying comparable infusion strategy to each patient if there was no massive hemorrhage. The data of postoperative HCT was acquired from the results of CBC test performed 24–36 h after surgery, thus there was enough time for fluid shift and volume balance [24]. Based on the recommendations by World Health Organization (WHO), primary PPH is defined as blood loss more than 500 ml within 24 h after delivery [25]. Since most peripartum hemorrhage occurs during or within the first 4 h posterior to delivery [26], the calculated blood loss measured 24–36 h postoperatively in this study is reflective of the above high-risk period. Although other methods could also be used in blood loss estimation, their limitations cannot be ignored. In our study, the visually estimated blood loss is less than the calculated value in both groups. This is consistent with the data in previous publications, indicating that the blood loss could be highly underestimated by visual estimation [27–29]. Gravimetric method has also been demonstrated low accuracy because of the invisible interference of the weight of amniotic fluid [30, 31]. A novel mobile application, photographic colorimetric system, has been proven to be more reliable, but it cannot be accessed in most countries [32, 33].

There were significantly more patients admitted to ICU in the thrombocytopenic group than in the control group. However, only two (16.7%) of them went to ICU for massive hemorrhage. In fact, most (9/12, 75%) of them had severe autoimmune disease with multi-organ involvement prior to surgery. It has been widely accepted that pregnancy could be a trigger for an increase in SLE activity [34]. Furthermore, thrombocytopenia in SLE patients during pregnancy is also a predictor for severe organ damage and poor prognosis [35]. Patients in the thrombocytopenic group also spent statistically more days in hospital after surgery, whereas the mean difference between groups was 0.40 day, which could be considered as clinically insignificant.

According to prior publications, well-recognized risk factors for excessive hemorrhage or blood transfusion of CS are: low BMI, prolonged operation duration, emergency, abnormal placentation, previous uterine scar, multiple pregnancies, neonatal macrosomia, placenta previa, placental abruption, preoperative anemia and general anesthesia [36–42]. In our study, we tried to control the confounders in three ways. First, we excluded patients with coagulopathy, platelet dysfunction, bleeding tendency, history of PPH, abnormal placentation and preoperative anemia. Second, the two groups were well-balanced in age and surgery timing (emergency or elective) at baseline by using matching approach. Third, there was significantly higher general anesthesia rate in the thrombocytopenia group than control group (22.6% vs 2.6%). In view of the possible association of general anesthesia with uterine atony and increased hemorrhage [43], anesthesia method may represent a significant confounder. Therefore, we included anesthesia method, along with BMI, operation duration and gestational weeks in the mixed-effect model. As a result, the upper bound of the two-side 95% CI of the calculated blood loss was still no greater than the pre-defined noninferiority delta.

Being observational and retrospective by nature, we cannot draw causal relationships and there could be inaccuracy in medical records. Furthermore, our study was subject to the following aspects of limitations. First, the calculated blood loss and transfusion rate is slightly lower than that reported previously [6, 13, 16], possibly due to the exclusion of patients with risk factors for excessive hemorrhage in our study. Consequently, the conclusion could not be hastily applied to patients at higher risk of massive blood loss. Additionally, since the average blood loss was within normal range in both groups, the potential effects of preoperative thrombocytopenia in patients who experienced primary PPH might be masked. To address this possibility, we conducted an exploratory subgroup analysis in patients with calculated blood loss > 500 ml. In this subgroup, the calculated blood loss was 679.7 ± 228.0 ml and 644.5 ± 175.7 ml in thrombocytopenic group (*n* = 34, 21.9%) and control group (*n* = 79, 25.5%), respectively. No significant difference was observed between groups (Student t-test *p* = 0.375). Second, considering a small sample size, the incidence of adverse event is very low and no hemostatic intervention or mortality was observed. Although we found no statistical differences between groups, perhaps there is still insufficient evidence to determine the impact of thrombocytopenia on these secondary outcomes. Third, our data was collected from a tertiary hospital, where patients were carefully evaluated during pregnancy, and the obstetrician team were experienced and skilled. Therefore, it is unknown whether our findings could also be instructive in primary medical settings with limited resources. Finally, we did not assess the clinical outcomes of neonates. Thus, the impact of maternal thrombocytopenia on infants’ PLT count at birth needs to be further explored.

## Conclusion

In conclusion, preoperative moderate thrombocytopenia (PLT 50–100 × 10^9^/L) does not increase the risk of blood loss, the requirement for blood transfusion, or the occurrence of adverse events in patients undergoing CS in absence of other risk factors for excessive hemorrhage. A restrictive preoperative PLT threshold might optimize blood utilization without worsening maternal outcomes. Prospective observational studies with larger sample size and randomized controlled trials are required to better refine the preoperative prophylactic platelet transfusion strategy.

## Data Availability

The datasets generated and analyzed during the current study are available from the corresponding author on reasonable request.

## Abbreviations

ASA: American Society of Anesthesiologists classification
BMI: Body mass index
CBC: Count of blood cell
CI: Confidence interval
CS: Cesarean section
DIC: Disseminated intravascular coagulation
FFP: Fresh frozen plasma
GA: General anesthesia
GT: Gestational thrombocytopenia
HCT: Hematocrit
HELLP: Hemolysis, elevated liver enzymes, low platelets
HGB: Hemoglobin
ICU: Intensive care unit
ITP: Idiopathic thrombocytopenic purpura
NA: Neuraxial anesthesia
PLT: Platelet
PPH: Postpartum hemorrhage
RBC: Red blood cell
SLE: Systemic lupus erythematosus
